# The intrinsic disorder challenge for AlphaFold: A case study of G3BP1 and pathogenic peptide

**DOI:** 10.1016/j.isci.2026.115737

**Published:** 2026-04-15

**Authors:** Yucong Li, Zhiying Yao, Zilin Song, Peiguo Yang, Jing Huang, Kai Lei, You Xu

**Affiliations:** 1 Fudan University, Shanghai, China; 2 State Key Laboratory of Gene Expression, School of Life Sciences, Westlake University, Hangzhou, Zhejiang, China; 3 Westlake Laboratory of Life Sciences and Biomedicine, Hangzhou, Zhejiang, China

**Keywords:** biological sciences, biochemistry, structural biology

## Abstract

The dipeptide repeat protein GR20 in amyotrophic lateral sclerosis (ALS) exerts neurotoxicity in part by binding to the stress granule protein G3BP1 and disrupting liquid-liquid phase separation (LLPS). However, the structural basis of this interaction remains elusive due to the pervasive intrinsic disorder in both partners. Here, we combine biochemical assays and structure prediction to characterize the G3BP1-GR20 complex. GR20 has high-affinity binding to G3BP1 and modulates LLPS in a concentration-dependent manner. Since the standard AlphaFold (AF) pipeline failed to predict credible models, we employed a constraint-based method AFEX to generate a G3BP1-GR20 complex model with improved confidence and structural plausibility. Our work underscores the necessity of extra efforts for AF predictions on disordered complexes and demonstrates the value of integrative and knowledge-guided approaches for exploring the “invisible proteome” of biomolecular condensates.

## Highlights

• AlphaFold fails to predict the disordered G3BP1-GR20 complex
• AFEX integrates biochemical knowledge to generate plausible structure
• NTF2L contributes to GR20 binding but is not the primary interaction driver
• Integrative modeling is essential for disordered proteins

## Graphical abstract

**Figure:**
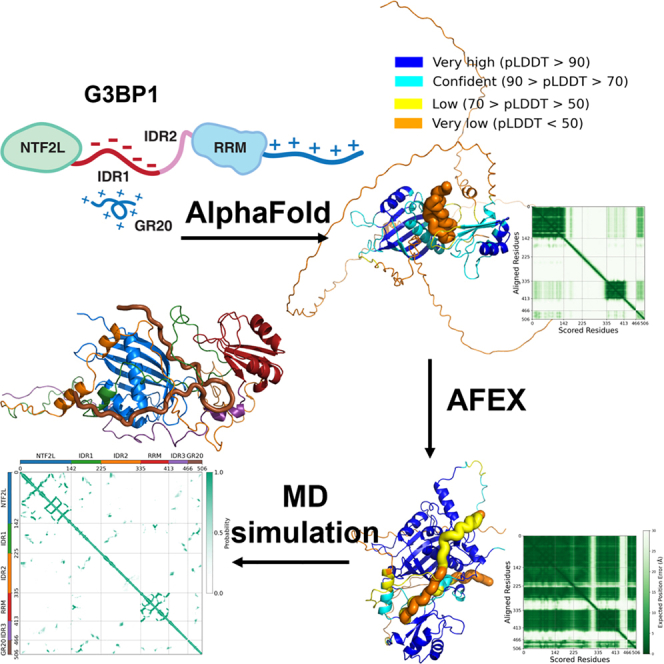

## Introduction

Amyotrophic lateral sclerosis (ALS) is a fatal neurodegenerative disorder characterized by the progressive degeneration of motor neurons.^1^^,^^2^^,^^3^^,^^4^ A major genetic cause of ALS as well as frontotemporal dementia (FTD) is the pathological expansion of a hexanucleotide repeat G4C2 in the *C9orf72* gene, which leads to the condition known as C9-ALS/FTD.^1^^,^^5^ One of its primary pathological mechanisms is the production of dipeptide repeat proteins (DPRs) through repeat-associated non-AUG translation.^6^^,^^7^^,^^8^^,^^9^ Among these, the arginine-rich DPRs, such as the glycine-arginine (GR), exhibit the highest toxicity.^10^^,^^11^^,^^12^ These inherently disordered peptides form pathological aggregates, disrupt key nuclear/cytoplasmic processes, and ultimately lead to neuronal dysfunction and death.^3^^,^^13^^,^^14^^,^^15^^,^^16^^,^^17^^,^^18^^,^^19^

**Figure 1:**
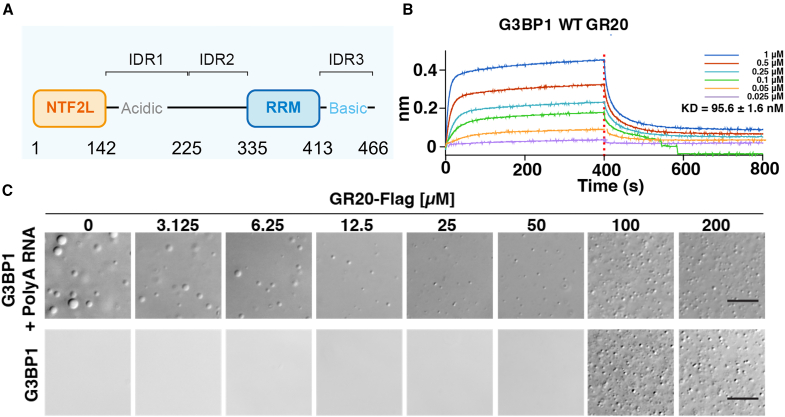
Domain architecture of G3BP1 and characterization of GR20 binding by BLI and LLPS assays (A) Schematic representation of G3BP1 domain organization. (B) BLI-binding curves for full-length G3BP1 with GR20. The dissociation constant was determined to be 95.6 ± 1.6 nM. The *x* axis represents time in seconds, while the *y* axis represents loading height in nanometers (nm). The lines in different colors indicate the GR20 concentrations. (C) The LLPS assay of full-length G3BP1 (50 μM) in the presence (upper) or absence (lower) of polyA RNA (100 ng/μL), across a range of GR20 concentrations (0–200 μM). Scale bars, 20 μm.

A critical process affected by the arginine-rich DPRs is the assembly and the dynamics of stress granules (SGs), which are membrane-less organelles formed through liquid-liquid phase separation (LLPS).^20^^,^^21^^,^^22^ SGs are regulated by multivalent interactions involving intrinsically disordered regions (IDRs) and low-complexity domains, and their dysregulation has been strongly implicated in neurodegenerative pathogenesis.^23^^,^^24^ One of the proteins central to the SG dynamics is G3BP1, a core scaffolding factor for SG assembly and is frequently perturbed in C9-ALS/FTD.^23^^,^^25^^,^^26^^,^^27^^,^^28^ G3BP1 is a multidomain protein featuring an N-terminal nuclear transport factor 2-like (NTF2L) domain, an RNA recognition motif (RRM) domain, as well as three IDRs (Figure 1A). Under normal conditions, G3BP1 adopts a closed conformation stabilized by intramolecular electrostatic interactions between its IDRs, which suppresses spontaneous phase separation.^28^ The NTF2L domain mediates critical homo-dimerization that is essential for facilitating G3BP1 function. Notably, the binding of DPRs to the negatively charged IDR1 disrupts the autoinhibitory state of G3BP1, promotes an open conformation, and drives aberrant LLPS, even more potently than RNAs.^29^ This DPR-induced malfunction of G3BP1 is a hallmark of ALS pathology.

Despite these insights, the structural mechanisms underlying G3BP1-DPR complex formation remain elusive, primarily due to the high degree of intrinsic disorder in both binding partners. The absence of experimental structural data for such complexes has made computational prediction an essential tool for mechanistic hypotheses. The advent of AlphaFold (AF) marked a revolutionary advance in structural biology, enabling highly accurate predictions of protein structures and interactions through deep learning (DL)-based modeling.^30^^,^^31^^,^^32^^,^^33^^,^^34^^,^^35^^,^^36^ Based on the transformative progress facilitated by the accurate structural predictions of protein single chains, complexes, and biomolecular interactions, the AF series of models has dramatically accelerated structural discovery. However, the AF models remain less reliable for IDR-containing proteins, which typically yield low-confidence predictions that reflect the highly dynamical and non-deterministic nature of these domains rather than their functional conformations.^37^^,^^38^ This limitation is particularly acute for systems such as G3BP1-GR20.

Numerous methods have been developed to postprocess or extend AF predictions to better capture the disordered, dynamical, and multiple conformations. For example, ColabFold improves the inference efficiency of AF through accelerated multiple sequence alignment (MSA)/template featurization and improved model compilation without compromising the predictive accuracy.^39^ Based on ColabFold, several approaches have been designed to exploit MSA subsampling for predicting alternative conformations of fold-switching proteins.^40^^,^^41^ Other tools integrate molecular dynamics (MD) simulations with AF-derived structures to access flexibility and refine structural stability, enabling advances in structural modeling and molecular design.^42^^,^^43^^,^^44^^,^^45^ Among these efforts, AFEXplorer (AFEX) is a computational framework designed to extend the utility of AF by generating protein conformations and biomolecule-relevant states that satisfy user-defined spatial constraints.^43^^,^^46^ By optimizing MSA featurization, AFEX introduces a conditioned prediction mechanism through introducing the concept of collective variables (CVs) constraints, which enables the integration of structural priors, such as inter-residue distances, secondary structure propensities, or functional domain orientations. A customizable loss function balances these constraints with structural confidence regularization, which guides the AF model toward physically plausible states relevant to expert-curated domain knowledge in biology.

In this study, we leverage the pathologically relevant G3BP1-GR20 complex to systematically evaluate the capabilities and the limitations of AF models in predicting highly disordered protein-peptide complexes. Combining biolayer interferometry (BLI) and phase separation assays, we establish the binding affinity behavior of GR20 with full-length G3BP1 and the isolated NTF2L domain. We then assess the performance of the standard AF protocols in recapitulating these interactions, which exposes the shortcomings of AF in handling intrinsic disorder. Finally, we explore the feasibility of extending the AF-Multimer protocol with AFEX to generate structurally informative and testable models. Our work showcases the integrative modeling for disordered proteins, highlighting the continued necessity of coupling computational predictions with experimental validation.

## Results

### GR20 binds to full-length G3BP1 and drives concentration-dependent phase separation

To establish the biochemical ground truth for the G3BP1-GR20 interaction, we performed biochemical assays. The BLI measurements confirmed a strong interaction between full-length G3BP1 and GR20 with a dissociation constant (*K*_*D*_) of 95.6 nM (Figure 1B). This affinity is approximately 15-fold weaker than that reported for the longer GR30 peptide in a previous study.^29^ Similar to GR30, GR20 also influenced SG assembly by modulating LLPS, though it required higher concentrations where the droplet formation initiated at 100 μM GR20 (Figure 1C). In the presence of RNA, GR20 modulated phase separation in a biphasic manner: it inhibited droplet formation at lower concentrations but promoted the liquid assembly at higher concentrations (Figure 1C). This behavior differs from that reported for GR30, where in a single concentration the LLPS is cooperatively enhanced when both DPR and RNA were present.^29^ These results suggest that, depending on specific conditions such as the DPR/RNA length and concentrations, GR20 may initially neutralize RNA before ultimately facilitating cooperative condensation.

### Standard AF protocols fail to predict the G3BP1-GR20 complex but suggest NTF2L-GR20 binding

**Figure 2:**
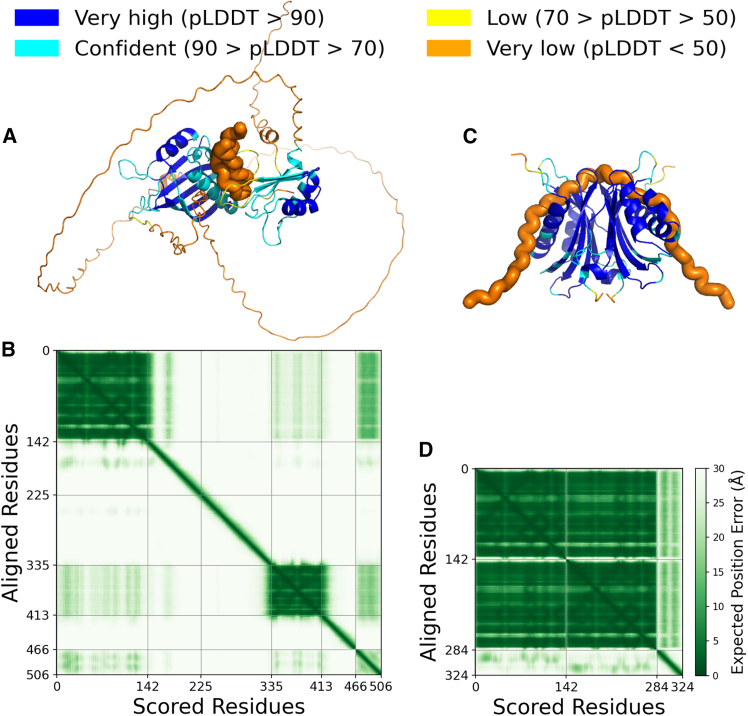
AlphaFold v.2.3 prediction of GR20 in complex with G3BP1 and the dimer of NTF2L domain (A and B) Complex of GR20 and G3BP1 monomer showing for (A) structure colored by residue-wise pLDDT scores and (B) its plot of PAE. (C and D) Complex of GR20 and NTF2L dimer showing for (C) structure colored by pLDDT scores and (D) its plot of PAE. Structures are aligned on the NTF2L domain. For structures showing as cartoon in (A and C), the GR20 peptides are highlighted with thicker coil, and the pLDDT color scale is indicated on the top.

Given the experimental evidence of binding affinity, we sought structural insights using the standard AF2 and AF3 protocols. However, both failed to generate reasonable complex structures for full-length G3BP1 monomer bound to GR20 peptide. The predicted models assigned high predicted local distance difference test (pLDDT) scores for residues in the structured NTF2L and RRM domains, whereas the IDRs and the DPR were highly unstructured with low scores (Figure 2A; Figure S1). No plausible interactions between G3BP1 and GR20 were observed. In AF2.2 and AF3, GR20 adopted an extended coil conformation without contacting G3BP1, while in AF2.3 it appeared as a collapsed coil on the NTF2L surface with steric clashes. The predicted alignment error (PAE) plots further indicated that relative residue positions were only reliable within structured domains, while inter-domain packing appeared essentially random (Figure 2B). Correspondingly, all models yielded very low multimer confidence scores (<0.4), indicating that AF could not converge to reliable binding poses (Table 1).This discrepancy stems from the prevalence of disordered regions in both binding partners and the absence of coupled folding and binding mechanisms.

**Table 1:** The metrics of AF- and AFEX-predicted models of G3BP1-GR20 and NTF2L-GR20 complexes

Metrics	G3BP1				NTF2L
	AF2.2	AF2.3	AF3	AFEX	AF2.3
Confidence	0.26	0.34	0.39	0.87	0.78
Ave pLDDT	54.6	51.4	64.8	80.6	82.5

We next isolated the structured NTF2L domain and predicted its complex with GR20 in the context of the functional homodimer. Although the GR20 region remained uncertain, overall confidence metrics improved markedly, with pLDDT and confidence scores exceeding 0.7 (Table 1). For example, AF2.3 predicted a well-structured NTF2L homodimer, where the two protomers associated through the hydrophobic β sheet interface. In this model, GR20 wrapped across the dimer interface, occupying regions adjacent to the canonical peptide-binding site of NTF2L (Figure 2C). This interpretation was supported by the PAE, which showed very low errors within the NTF2L dimer but high errors for regions involving GR20 (Figure 2D). The predicted binding interface aligns with the reported sites of both NTF2^47^ and NTF2L,^48^ including the loops adjacent to the β sheets and the hydrophobic groove between the α helices.

### NTF2L domain binds GR20 with mild affinity but is not the primary-binding site of G3BP1

**Figure 3:**
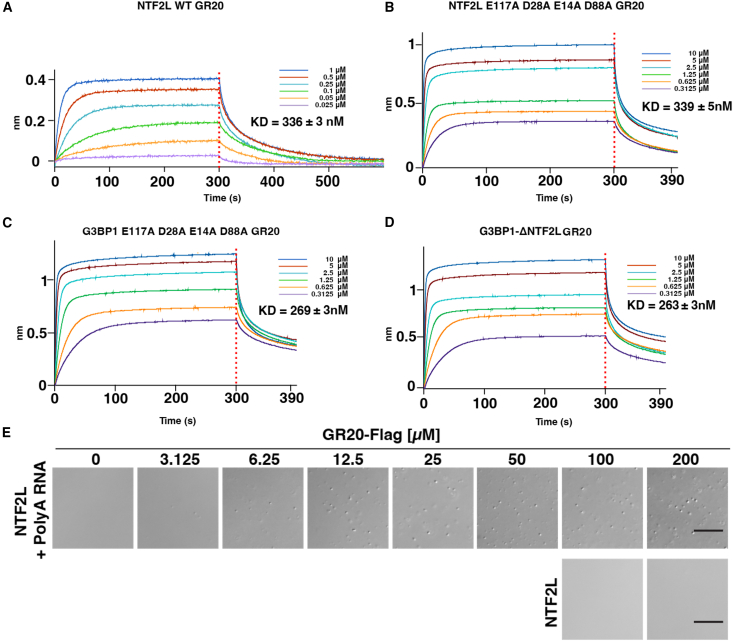
Binding affinity and phase separation behavior of GR20 with the NTF2L domain and G3BP1 variants (A) BLI-binding curve the isolated NTF2L domain with GR20, *K*_*D*_ = 336 ± 3 nM. (B) Binding of the NTF2L domain carrying quadruple alanine mutations (E14A, D28A, D88A, and E117A) with GR20, *K*_*D*_ = 339 ± 5 nM. (C) Binding of full-length G3BP1 carrying the same quadruple mutations with GR20, *K*_*D*_ = 269 ± 3 nM. (D) Binding of G3BP1 lacking the NTF2L domain (ΔNTF2L) with GR20, *K*_*D*_ = 263 ± 3 nM. (E) The LLPS assay with the isolated NTF2L domain (50 μM) in the presence (upper) or absence (lower) of polyA RNA (100 ng/μL), across a range of GR20 concentrations (0–200 μM, upper, and 100–200 μM, lower). Scale bars, 20 μm.

To experimentally assess the AF2 predictions, we measured the binding affinity between the isolated NTF2L domain and GR20 using BLI. The NTF2L domain bound GR20 with a *K*_*D*_ of 336 nM, approximately 3.5-fold weaker than that of G3BP1 (Figure 3A). Furthermore, the faster saturation and dissociation kinetics suggest that NTF2L-GR20 binding is less specific and thermodynamically less stable.

To examine how acidic residues in NTF2L contribute to DPR binding, we introduced a quadruple mutation (E14A, D28A, D88A, and E117A) in both the isolated NTF2L domain and full-length G3BP1. These residues are located in known binding interfaces and were also implicated in AF-predicted models (Figure S2A). The mutations did not alter binding affinity in the isolated NTF2L domain (339 nM vs. 336 nM) but led to a modest reduction in full-length G3BP1 (269 nM vs. 96 nM) (Figures 3B and 3C). Since arginine can engage in π-π interactions,^49^^,^^50^ we also introduced two single mutations (R17K and R32K) at the binding interface to remove the π-electron-rich guanidinium group. These mutations similarly resulted in negligible changes in binding affinity in full-length G3BP1 (Figures S2B and S2C).

The NTF2L domain mediates G3BP1 dimerization that is essential for RNA-triggered LLPS, where the NTF2L acts as a structural scaffold rather than a specific binding interface.^28^ Although we did not assess the role of NTF2L in DPR-induced LLPS, deletion of the entire NTF2L domain led to a binding affinity comparable to that of the quadruple mutant (263 nM vs. 269 nM) (Figure 3D). As expected, the isolated NTF2L domain was unable to initiate LLPS even at high GR20 concentrations (Figure 3E). In the presence of RNA, small droplets emerged as GR20 concentration increased, likely reflecting condensates formed between RNA and DPRs rather than involving NTF2L.

These results indicate that in the full-length G3BP1, the NTF2L domain contributes to GR20 binding but is not the primary driver of direct interactions. In the isolated form, NTF2L-DPR binding appears to be a kinetically rather than thermodynamically driven process, with no specific binding pose or pocket maintained. Collectively, these findings suggest that within G3BP1, the NTF2L domain facilitates GR20 binding not through a conserved binding mode, but through contextual mechanisms involving direct electrostatic contact, structural positioning, and allosteric coupling with IDRs mediated by dimerization.

### AFEX yields improved model for the G3BP1-GR20 complex

The discrepancy between the experimental data and the standard AF predictions suggests that AF tends to assign high confidence to structured domains while struggling with IDRs due to their low sequence conservation. To address this, we employed the AFEX protocol, an extended algorithm that incorporates structural restraints and sampling strategies tailored for disordered systems. Customized CVs were defined between IDR1 and GR20 and between IDR1 and IDR3 (see STAR Methods), to guide structural optimization toward known domain contact. The loss function incorporated CVs and regularization metrics to enable holistic model refinement.

**Figure 4:**
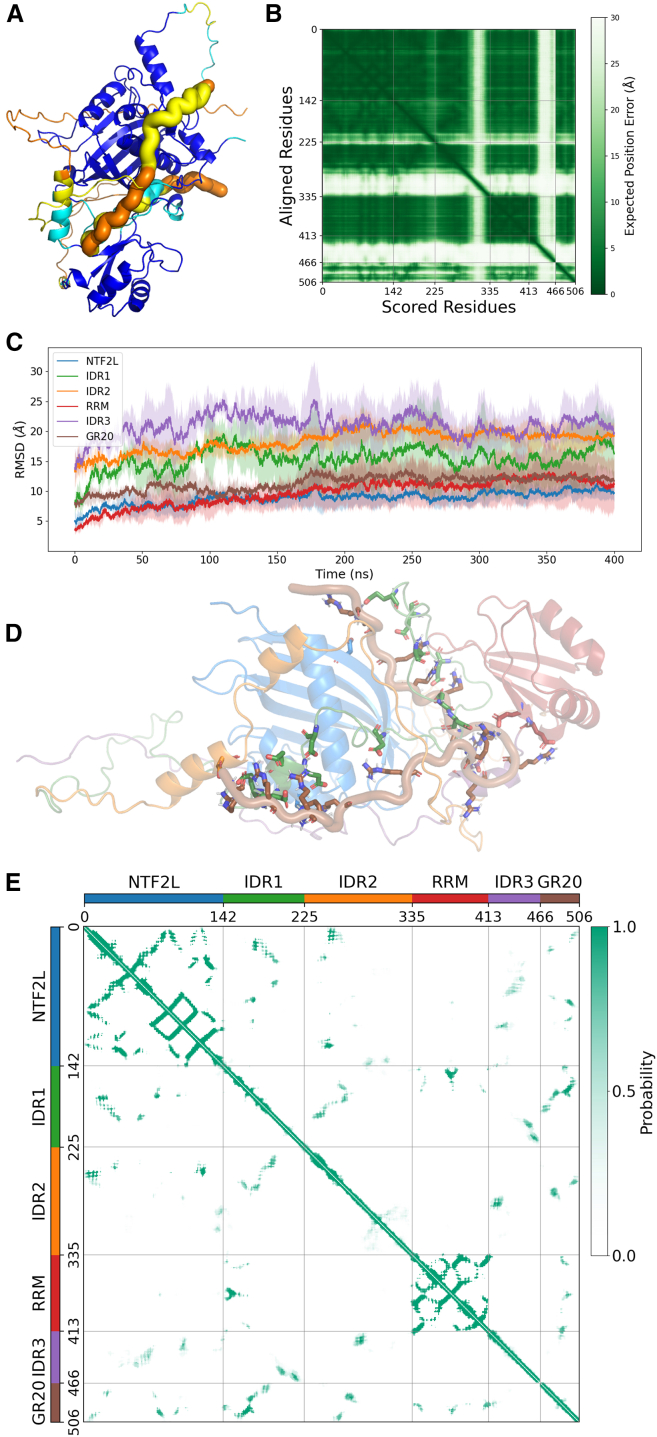
AFEX predicted G3BP1-GR20 complex and its features in MD simulations (A and B) The AFEX-generated model for (A) structure colored by pLDDT scores and (B) its plot of PAE. The structural representation, orientation, and color scheme are same as in Figure 2. (C) Heavy-atom root-mean-square deviation (RMSD) relative to the initial AFEX model over MD simulation time. Transparent shading represents the standard deviation of values from three independent simulation replicates. (D) Average structure over all trajectories of MD simulations. The structure is in transparent cartoon with GR20 highlighted with thicker coil. The interactive Arg in GR20 and Asp/Glu in G3BP1 are shown in sticks. (E) Residue contact map represented by pairwise Cα distance. The probability is counted from all trajectories. (C)–(E) adopt the same color scheme for structural domains: marine for NTF2L, green for IDR1, orange for IDR2, crimson for RRM, violet for IDR3, and brown for GR20.

Starting from the AF-predicted models, AFEX generated full-length G3BP1-GR20 complexes with significantly improved residue-wise pLDDT scores (>80) (Figure 4A). The AFEX model featured the formation of short α helices in IDR1 and IDR2, which anchored to the α helices of NTF2L, substantially increasing local pLDDT, while leaving the remaining regions disordered. GR20 was positioned in extensive contact with the coil of IDR1 and other regions of G3BP1, situated at the interface between the structured NTF2L and RRM domains. Correspondingly, the PAE was markedly reduced not only for residues aligned on structured domains but also for the ones aligned on some part of IDR1 and IDR2 (Figure 4B), which indicates a more reliable inter-residue positioning for these regions. The more compact organization led to a notable increase in the multimer interaction confidence score (>0.8) (Table 1). Such architecture is mechanistically plausible, as it aligns with the known role of IDR1 upon DPR binding.

To assess the thermal stability of the predicted structure, we performed MD simulations on the AFEX-derived model at 298 K and 1 bar. The complex exhibited large-scale fluctuations, consistent with the expected dynamics of an IDR-containing system. The structured NTF2L domain showed the lowest heavy-atom root-mean-square deviation (RMSD) (7 Å), followed by the RRM domain (9 Å), and the GR20 trajectories displayed RMSD values (10 Å) roughly close to them (Figure 4C). The flexibility of IDR1 was also reduced (14 Å) relative to IDR2 (19 Å) and IDR3 (22 Å), which reflects extensive involvement in GR20 interactions. The IDR2 formed stable helices contacting NTF2L and was less flexible than IDR3; the latter had limited interactions and more solvent exposure. At elevated temperatures (350–500 K), RMSD values for GR20, IDR1, and IDR2 increased substantially (Figure S3). At 420 K and 500 K, all domains showed further RMSD increases beyond 400 ns, indicating thermal destabilization, which is consistent with the expected behavior of a dynamic IDR complex.

Throughout the simulations, most arginine residues of GR20 engaged in persistent interactions with aspartic and glutamic acids primarily located in IDR1, along with a few from other domains (Figure 4D), which support the hypothesis that it is the cumulative electrostatic attraction that dominates the interaction. This agrees with previous studies where charge-reversal mutations in IDR1 abolished LLPS, while randomizing the IDR1 sequence (preserving composition) or deleting IDR2 or IDR3 had no obvious effect.^28^^,^^29^ Notably, the newly formed short α helices in IDR1 and IDR2 remained stable and packed against NTF2L during simulations, which demonstrates the capability of AFEX combined with MD simulations to identify thermodynamically stable states in IDR-containing complexes.

The residue-contact analysis of MD trajectories reveals persistent interaction patterns throughout the simulation. The AFEX-generated model not only enabled interactions between GR20 and G3BP1, especially IDR1 but also increased the contact chances among G3BP1 domains themselves (Figure 4E). Specifically, IDR1 established contacts with all other domains, consistent with its central role in GR20 binding. IDR2 contacted all domains except RRM, whereas IDR3 showed no contacts with GR20 due to the repulsive restraints applied in AFEX. GR20 itself exhibited broad interactions with all domains except IDR3, reflecting its engagement with electrostatic interactions. This pattern aligns with the RMSD analysis: domains with higher contact densities displayed lower RMSD values, indicating reduced conformational fluctuation through stronger interactions. In contrast, standard AF-predicted structures showed only sparse inter-domain contacts restricted to the NTF2L domain with essentially no contacts involving IDRs (Figure S4). The rich contact network captured by the AFEX-MD models characterizes IDR-mediated assemblies with multivalent, dynamic, and redundant interactions, which is distinct from the induced-fit binding in folded proteins.^51^

In summary, while we could not present the prediction as a definitive structure, the AFEX model provides a testable hypothesis that GR20 binding involves a multimodal mechanism engaging both structured and disordered regions. This is an insight entirely absent from standard AF predictions.

## Discussion

Arginine-rich DPRs play a central role in the neurotoxicity associated with C9-ALS/FTD, with their pathogenicity strongly correlating with intrinsic structural disorder. Our results reveal that the standard AF protocols failed to produce reliable structural models for the G3BP1-GR20 complex, despite the experimental evidence of strong binding. Although AF shows higher confidence when predicting the interaction between GR20 and the structured NTF2L domain, these predictions reflect only part of the biochemical evidence: the isolated NTF2L domain binds GR20 mildly and cannot recapitulate the strong affinity and phase separation behavior observed with full-length G3BP1. This discrepancy underscores a fundamental limitation of current protein structure predictors in modeling complexes involving highly disordered regions.

This challenge is unlikely to be unique to G3BP1-GR20. As research increasingly focuses on the “invisible proteome” with intrinsically disordered regions, the inability of AF to model dynamic and multivalent interactions will become more apparent. Our work serves as a cautionary note that the AF predictions with low-confidence scores should not be dismissed as non-interacting without experimental validation. Conversely, even high-confidence predictions of binding sites within structured domains, such as those observed with NTF2L-GR20 complex, should be interpreted as hypothetical rather than definitive, particularly when the multimerization context involves disordered regions. This limitation extends beyond IDR-containing systems: AF also fails to predict alternative conformations for the vast majority of experimentally characterized fold-switching proteins, despite the strong sequence conservation.^52^^,^^53^ These proteins encode coevolutionary signatures for both folds but conventional AF pipelines capture only one dominant state, which is related to the challenges encountered in this study. Alternative models that perform well for local dynamics show complementary strengths yet tend to over-stabilize the IDR-rich systems. For instance, our tests with Boltz-2^54^ displayed excessive helices and sheets within IDRs for G3BP1-GR20, yielding structures that resemble folded proteins (Figure S5).

AFEX represents a form of integrative modeling, blending a data-driven DL model with empirical restraints based on knowledge of experimental data. In this study, binding affinities and electrostatic properties guided the selection of CVs, such as the attraction between the acidic IDR1 and the basic GR20/IDR3, and the repulsion between GR20 and IDR3. This approach yielded a full-length complex model with improved confidence scores and a physically reasonable architecture involving both structured and disordered regions. By integrating domain-specific knowledge into AF predictions, it generates alternative conformations for further exploration of the conformational space. The AFEX strategy can potentially be extended to other biological foundation models for co-folding protein complexes. As a flexible framework for diverse protein systems, AFEX performance depends heavily on prior biochemical knowledge (e.g., identification of interacting regions), and the choice of CVs can be subjective. Although AFEX produces models that satisfy predefined CVs and exhibit higher formal confidence metrics (i.e., pLDDT, pTM, and ipTM), the resulting structures should be interpreted as plausible conformational snapshots rather than (free) energy minima or thermodynamical averages, unless the representative ensemble is subsequently explored through extensive MD simulations.

AFEX is not specifically optimized for disorder-to-order transitions, but its conditioning framework can be adapted to model such cases if appropriate CVs are defined. Nevertheless, several limitations warrant consideration. First, as its performance is inherently limited by the underlying AF-Multimer architecture, AFEX tends to guide structures toward canonical protein-like architectures. For example, it promotes helix formation in non-Gly/Pro context of IDR1 and IDR2 of G3BP1 to enhance the inter-domain contacts. While the conformations preserved stability in MD simulations, they may not fully represent the dynamic reality of the native disordered complexes. Second, AFEX does not incorporate energy functions so it cannot explicitly avoid steric clashes or enforce energetic validity. Akin to the original AF inference pipeline, the AFEX-conditioned structural outputs require energy minimization using force fields. Consequently, the thermodynamic calculations such as binding affinity estimation remain a post-processing task better addressed by MD-based methods.

Accordingly, several strategies can be proposed to strengthen the applicability of AFEX-related approaches. First, integrating more automated and biophysically informed constraints, such as small-angle X-ray scattering data, NMR chemical shifts, or mutational scanning data, could reduce reliance on manual CV selection and improve the modeling of biological relevance. Second, a combined protocol that uses AFEX to generate initial conformations followed by extensive MD simulations would help validate structural stability and discover more representative ensembles. AFEX provides valuable starting points for simulations, which is essential for studying disordered proteins. At least for now, the hybrid approaches that leverage both DL and physics-based modeling represent a practical and promising solution.

In summary, this study presents a multifaceted analysis of the ALS-associated G3BP1-GR20 interaction through integrated biochemical characterization and computational modeling. Our findings demonstrate that while standard AF has revolutionized structural prediction for ordered systems, it remains inadequate for highly disordered complexes, as low-confidence AF outputs fail to recapitulate the high binding affinity and phase separation behavior observed experimentally. AFEX is a constraint-based extension that incorporates prior knowledge to refine AF predictions in handling IDRs and interface loops, which yields structurally plausible and testable hypotheses. Although AFEX does not directly elucidate biological mechanisms, it provides user-refined structures incorporating experimental evidence as starting points, which can inform future mechanistic studies.

This work reinforces that blind reliance on AI predictions is insufficient for exploring the “invisible proteome”. Future advances will require the pragmatic integration of DL with experimental data and physical constraints. Approaches like AFEX represent a step in this direction, which offers a customizable framework to build testable models of biologically critical and therapeutically relevant complexes. By openly addressing the limitations of end-to-end DL and promoting integrative strategies, we illustrate a more adaptable and empirically grounded paradigm for studying biomolecular condensates. Continued refinement of such methods will likely enable deeper insights into the mechanism of G3BP1-DPR interactions in ALS.

### Limitations of the study

First, AFEX relies on user-defined CVs derived from prior biochemical knowledge, which introduces a degree of subjectivity and may limit its applicability where such information is unavailable. Second, the method does not incorporate an explicit energy function, so the structural outputs require subsequent energy minimization and MD simulations to assess stability and sample conformational ensembles. Third, although AFEX improved confidence metrics, its performance is inherently constrained by the underlying AlphaFold-Multimer architecture, which tends to bias predictions toward canonical protein structures. Fourth, our MD simulations, while informative, may not fully capture the conformational landscape of such highly dynamic systems.

## Resource availability

### Lead contact

Further information and requests for resources and reagents should be directed to and will be fulfilled by the lead contact, You Xu (xuyou@westlake.edu.cn).

### Materials availability

All plasmids and reagents generated in this study are available from the authors upon reasonable request.

### Data and code availability

• The AFEX code: https://github.com/JingHuangLab/AFEXplorer-min.
• Protein sequences were obtained from UniProt. The server/packages are AlphaFold (https://deepmind.google/technologies/alphafold/), Boltz-2 (https://app.tamarind.bio/boltz), PyMOL (https://pymol.org/), CHARMM (https://www.academiccharmm.org/program), OpenMM (https://openmm.org/), and MD Analysis toolkit (https://www.mdanalysis.org/).
• Additional information will be available from the lead contact upon request.

## Acknowledgments

Y.X. thanks Dr. Tengyu Xie for useful discussions and Kunying Niu for Boltz-2 implementation. We thank Ping Zhang, Jiao Liu, and Huisong Ma for assistance with the BLI experiments. The High-Performance Computing Center and the Protein Characterization and Crystallography Facility of Westlake University are acknowledged for technical support. This work was supported by the 10.13039/501100012166National Key R&D Program of China (2025YFC3408900 to K.L.), the “Pioneer” and “Leading Goose” R&D Program of Zhejiang (2023C03109, 2024SSYS0030 to J.H.), the Key Research Program of Zhejiang (QKWL25H0901 to K.L.), the 10.13039/501100001809National Natural Science Foundation of China (T2596084 to J.H. and 22503068 to Z.S.), the Hangzhou Joint Fund of 10.13039/501100004731Zhejiang Provincial Natural Science Foundation of China (LHZQN26B030001 to Z.S.), the Zhejiang Provincial Key Laboratory Construction Project, the State Key Laboratory of Gene Expression, the 10.13039/100032572Westlake Education Foundation, the ICBC “Junzi Fellowship Charitable Trust”, and the Hangzhou Leading Innovation Team (TD2024001).

## Author contributions

Y.X., K.L., Y.L., and J.H. conceived the project, designed experiments, and wrote the manuscript. Y.L. performed AF prediction, protein purification, and BLI assays. Z.Y. and P.Y. conducted the data acquisition and analysis of LLPS experiments. Z.S. developed the AFEX code. Y.X. performed the AFEX and the MD simulations.

## Declaration of interests

The authors declare no competing interests.

## STAR★Methods

### Key resources table

**Table:** 

REAGENT or RESOURCE	SOURCE	IDENTIFIER
**Bacterial and virus strains**		

*Escherichia coli* DH5α	Kangti Life Sciences	N/A
*Escherichia coli* BL21(DE3)	Kangti Life Sciences	N/A

**Chemicals, peptides, and recombinant proteins**		

GR20 peptide	GL Biochem	N/A
PR20 peptide	GL Biochem	N/A
Poly(A) RNA	Sigma	Cat#1018626001
Pierce™ Glutathione Agarose	Thermo Scientific	Cat#78601
Reduced glutathione	Sangon Biotech	Cat#70-18-8
Isopropyl β-D-1-thiogalactopyranoside (IPTG)	Inalco	Cat#1758-1400
Ampicillin sodium	Sangon Biotech	Cat#69-52-3
Phenylmethylsulfonyl fluoride (PMSF)	Thermo Fisher Scientific	Cat#36978
Protease inhibitor cocktail	Roche	Cat#11873580001
Octet Anti-GST biosensors	ForteBio	Cat#18-5160

**Critical commercial assays**		

Gel Extraction Kit	GenStar	Cat#D205-04
Plasmid Mini Kit	Vazyme	Cat#DC201-01

**Recombinant DNA**		

pGEX-6P-1-hsG3BP1-WT	this paper	N/A
pGEX-6P-1-hsG3BP1-E14A/D28A/D88A/E117A	this paper	N/A
pGEX-6P-1-hsG3BP1-R32K	this paper	N/A
pGEX-6P-1-hsG3BP1-R17K	this paper	N/A
pGEX-6P-1-hsG3BP1-ΔNTF2L	this paper	N/A
pGEX-6P-1-hsNTF2L-WT	this paper	N/A
pGEX-6P-1-hsNTF2L-E14A/D28A/D88A/E117A	this paper	N/A

**Software and algorithms**		

AlphaFold	DeepMind	https://deepmind.google/technologies/alphafold/
AFEXplorer	Jing Huang Lab	https://github.com/JingHuangLab/AFEXplorer-min
CHARMM	CHARMM Development Project	https://www.academiccharmm.org/program
OpenMM	OpenMM	https://openmm.org/
MDAnalysis	MDAnalysis	https://www.mdanalysis.org/

**Other**		

Octet RED96 system	ForteBio	N/A

### Experimental model and study participant details

#### Bacterial strains

Recombinant protein expression was performed in *Escherichia coli* BL21(DE3) cells grown in LB medium at 37°C unless otherwise specified.

### Method details

#### Protein structure prediction and visualization

The protein complex structures were predicted using AlphaFold-multimer models v2.2, v2.3, and AlphaFold 3.^30^^,^^31^ The full-length sequence (466 aa) of G3PB1 was downloaded from UniProt (https://www.uniprot.org/), and the NTF2L domain is the first 142 aa of G3BP1. Outputs were evaluated using the predicted local distance difference test (pLDDT) score, the predicted TM-score (pTM), and the interface predicted TM-score (ipTM). The structures were ranked using the confidence score of multimer, which is calculated as 0.8 × ipTM + 0.2 × pTM. The one with the highest score of each AF model was selected to present, and the one predicted by AF v2.3 was also the initial structure for further AFEX optimization. Structural visualizations were performed using PyMOL v3.1.6.

#### AFEX loss function and CV selection

AFEX was implemented on AF multimer v2.3, where the algorithm optimizes protein structures through a custom loss function $L^{\text{AFEX}}$, which is defined as:

$$L^{\text{AFEX}}={w_{c}*L}^{\text{CV}}+{w_{r}*L}^{\text{regular}}$$

where **w** represents the array of weights. The regularization term $L^{\text{regular}}$ incorporates the standard AF-Multimer confidence metrics:

$$L^{\text{regular}}=1-\text{mean}\left(y^{\text{pLDDT}}\right)+1-\text{mean}\left(y^{\text{pTM}}\right)+1-\text{mean}\left(y^{\text{ipTM}}\right)$$

where **y**^pLDDT^, **y**^pTM^, and **y**^ipTM^ represent the pLDDT, pTM, and ipTM scores, respectively, and then averaged over all atoms. The collective variable term $L^{\text{CV}}$ consists of structure-restrained, attractive and repulsive components:

$$L^{\text{CV}}=L^{\text{rest}}+L^{\text{attr}}+L^{\text{repl}}$$

With:

$$L^{\text{rest}}=\text{mean}\left(\sum_{i}\sqrt{{\left(r_{i}-r_{i,\text{ref}}\right)}^{2}}\right)$$

$$L^{\text{attr}}=\max\left(\text{mean}\left(d-d_{c}\right),0\right)$$

$$L^{\text{repl}}=\max\left(\text{mean}\left(d_{c}-d\right),0\right)$$

Here, **r**_***i***_ denotes the array of Cα cartesian coordinates of AFEX outputs for only structured domains (NTF2L and RRM in this study), and **r**_***i***,ref_ is the array of those coordinates predicted by AF; **d** denotes the array of Cα distances between selected residue groups, and **d**_*c*_ is the distance cutoff, uniformly set to 8 Å in this study. Attractive restraints were applied between the following residue groups to promote proximity between IDR1 and GR20: residues [174, 187, 167, 163, 154] and [477, 475, 493, 495, 501]; and between IDR1 and IDR3: residues [202] and [452]. A repulsive restraint was applied within GR20 (residues [469, 473, 478, 474] and [503, 492, 498, 502]) to prevent unnatural peptide self-aggregation. Such residue selection was empirically guided and adapted based on the AF output in use to enhance physical plausibility. During AFEX optimization, the weight of CV term **w**_**c**_ was set to 1 to strongly enforce spatial constraints and was gradually reduced to 0.1. Conversely, the weight of the regularization term **w**_**r**_ began at 0 and was incrementally increased to 1, to allow the model to evolve toward conformations that balance user-defined restraints with structural confidence.

#### MD simulations and analysis

MD simulations were conducted on the AFEX generated complexes G3BP1-GR20. Preliminary minimization was conducted in vacuum first using a set of decreased harmonic restraint forces from 100000 to 5 kcal/mol on the backbones of NTF2L and RRM domains, to relax the atomic clashes gradually. The systems were then solvated in a 107 × 107 × 107 Å^3^ cubic TIP3P water box, neutralized with sodium ions, and maintained in 0.15 M NaCl. System preparation was carried out using the CHARMM software suite^55^ with the CHARMM36m protein force field.^56^^,^^57^

Simulations were run using the OpenMM platform.^58^ Prior to production, each structure underwent a 5 ns equilibration, during which positional restraints were gradually reduced from 5 kcal/mol to zero, and the integration timestep was increased from 1 fs to 2 fs. The production phase used the velocity Verlet integrator in *NpT* ensemble at 298 K and 1 bar, using a Langevin thermostat and a Monte Carlo barostat. To investigate the thermal stability, higher temperatures 350 K, 420 K and 500 K were also adopted. Each independent run lasted 400 ns and totaling 1200 ns. Trajectories were recorded at 200 ps intervals. Analyses were performed using the CHARMM program and the MDAnalysis toolkit.^59^ Residue contacts were defined based on a Cα–Cα distance cutoff within 12 Å. For the standard AF-predicted models, contact probabilities were calculated and averaged across all relaxed output structures. For the AFEX-refined model, contact frequencies were determined by summing all contacts observed during three independent MD trajectories and then averaging across them.

#### Biolayer interferometry (BLI) assays

BLI assays were performed using the Octet RED96 system (ForteBio). GST-tagged G3BP1 and NTF2L (0.5 μM) were immobilized onto Dip and Read Anti-GST biosensors and equilibrated in a buffer containing 150 mM NaCl, 50 mM Tris-HCl, and 0.05% Tween at pH 7.4. The biosensors were then exposed to varying concentrations of GR20 and PR20 peptides, followed by a dissociation phase in the same buffer. Binding affinity (*K*_D_) was determined using ForteBio Data Analysis software. For sequential binding analysis, biosensors loaded with G3BP1 or NTF2L were first exposed to 0.5 μM G3BP1 or NTF2L, re-equilibrated, and then incubated with GR20 or PR20 peptides before a final wash. Non-specific binding controls were included to ensure specificity.

#### Mutagenesis and protein preparation

Site-directed mutagenesis was performed using a PCR-based method. All constructs were verified by Sanger sequencing. The following constructs were cloned into the pGEX-6P-1 vector with an N-terminal GST tag: full-length human G3BP1 (wild-type), and its quadruple mutant (E14A, D28A, D88A, E117A), and single mutants R32K and R17K; an NTF2L-domain knockout mutant of G3BP1; the isolated NTF2L domain (wild-type) and its quadruple mutant (E14A, D28A, D88A, E117A). Each plasmid was transformed into BL21(DE3) *E. coli* cells. Cultures were grown at 37 °C until OD600 reached 0.8–1.0, then induced with 1 mM IPTG and incubated at 16 °C for 16 h with shaking. Cells were harvested and resuspended in lysis buffer (50 mM Tris-HCl, pH 7.4–7.6, 400 mM NaCl), followed by sonication and centrifugation to remove insoluble material. The clarified lysate was loaded onto glutathione agarose resin. The resin was washed with at least five column volumes of equilibration buffer (same as the lysis buffer). Target proteins were eluted with equilibration buffer containing 10 mM reduced glutathione, collected in fractions, quantified by a Coomassie G-250 assay, concentrated, flash-frozen, and stored at -80°C.

#### Liquid-liquid phase separation

LLPS experiments were conducted at room temperature unless stated otherwise. PolyA RNA (1 mg/mL, Sigma, Cat#1018626001) was prepared in 50 mM Tris-HCl (pH 7.6), aliquoted, and stored at -80°C. G3BP1 and DPRs were diluted from 400 mM to 300 mM salt buffer in 50 mM Tris-HCl (pH 7.6). Equal volumes of DPRs and polyA RNA were mixed to achieve a final 150 mM salt concentration. The mixtures were pipetted three times in a 0.2 mL PCR tube, transferred to a chamber slide (cover glass and slide separated by double-sided tape), and observed under a DIC microscope (40X lens). Images were captured within 5 minutes after phase separation induction.

For the three-component LLPS assay, G3BP1 and DPRs were diluted to the desired concentrations in 300 mM NaCl and 50 mM Tris-HCl (pH 7.6). Equal volumes of 200 μM G3BP1 and either control buffer or DPRs were mixed to maintain 300 mM NaCl. Then, an equal volume of 200 ng/mL polyA RNA was added to trigger phase separation. The samples were prepared as above and observed under a microscope.

### Quantification and statistical analysis

MD simulation systems were performed with three independent runs, and the data were reported as average with standard deviation.

### Additional resources

No additional resources are available.
