# Supplementary material for: The intrinsic disorder challenge for AlphaFold: A case study of G3BP1 and pathogenic peptide
Source: iScience. 2026 Apr 15;29(5):115737. doi: 10.1016/j.isci.2026.115737 (PMC13157106; doi:10.1016/j.isci.2026.115737)
Supplement: Document S1. Figures S1–S5 [file mmc1.pdf]

iScience, Volume *29*

Supplemental information

The intrinsic disorder challenge for AlphaFold: A case study of G3BP1 and pathogenic peptide

Yucong Li, Zhiying Yao, Zilin Song, Peiguo Yang, Jing Huang, Kai Lei, and You Xu

Supplemental figures

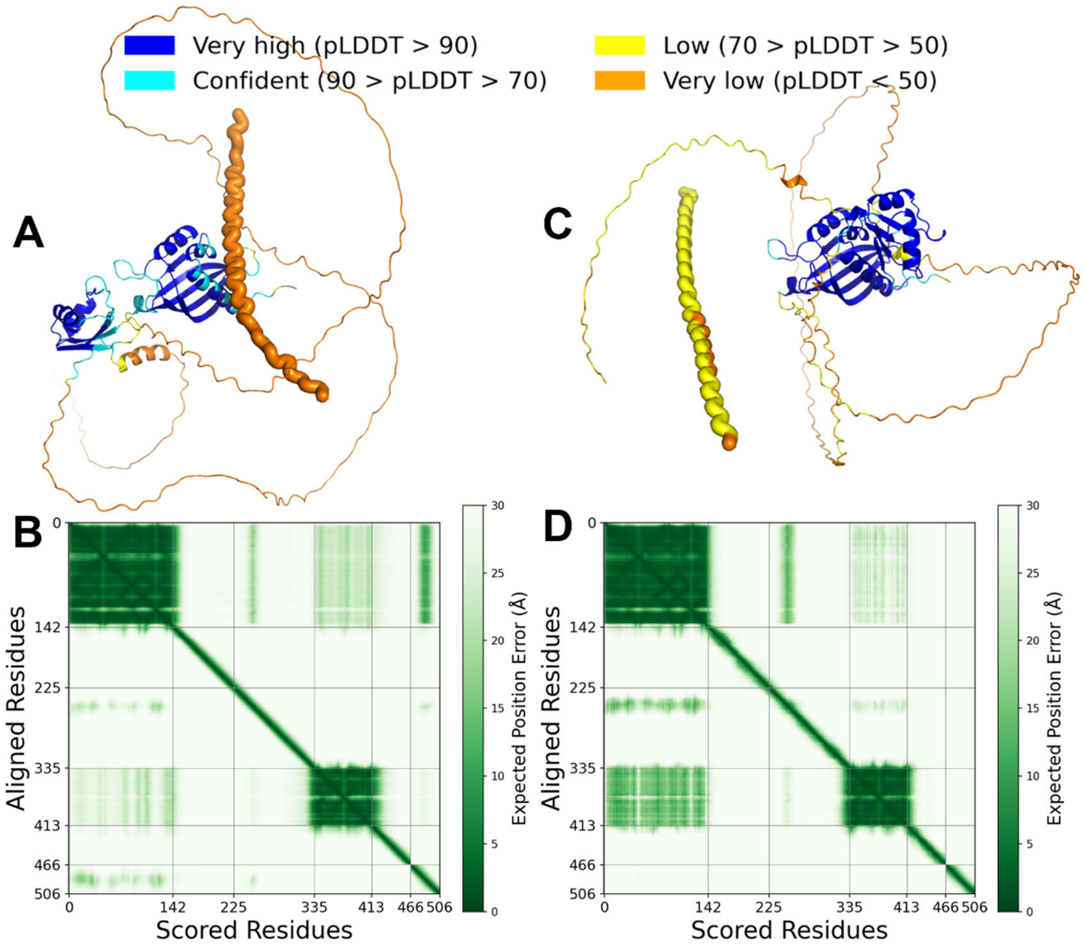

Figure S1. AlphaFold-predicted models of GR20 in complex with G3BP1. Predicted structures of the G3BP1 monomer in complex with GR20 generated using (A-B) AlphaFold v2.2 for (A) structure colored by residue-wise pLDDT scores and (B) its plot of predicted aligned error (PAE); and (C-D) AlphaFold v3 for (C) structure colored by pLDDT and (D) plot of PAE.

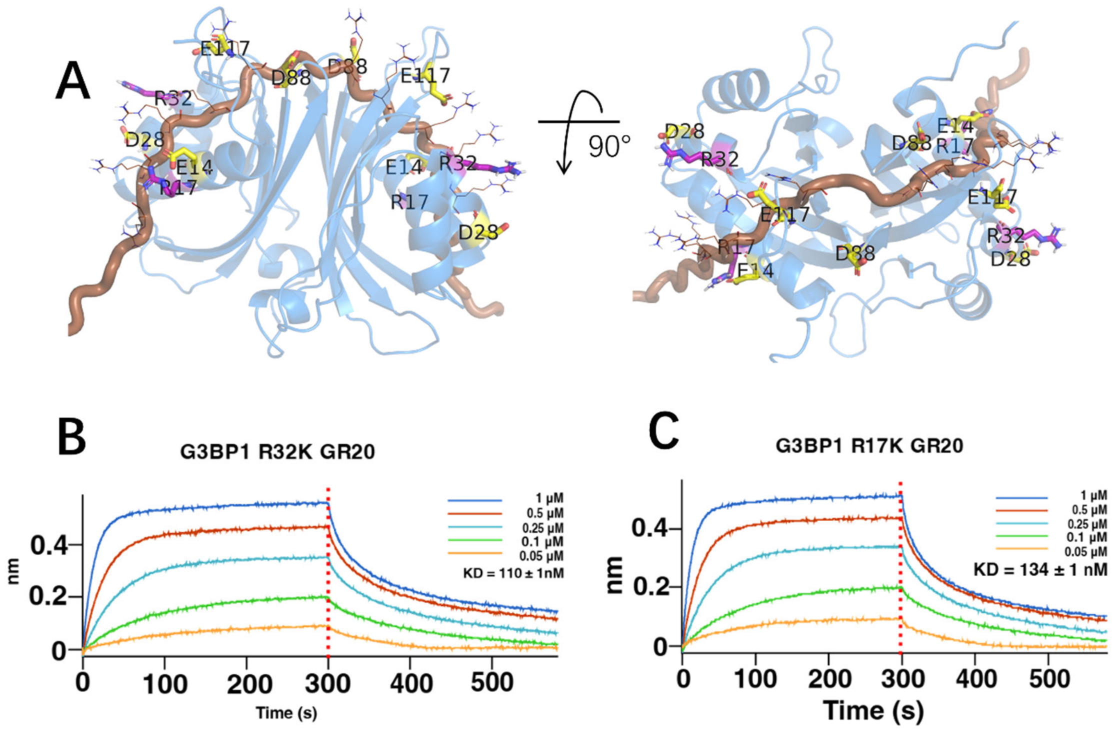

Figure S2. AF predicted GR20 binding interface of the NTF2L domain and binding affinity of two single mutations. (A) Complex of GR20 and NTF2L dimer showing the interface acidic residues and arginine. The structure is as transparent cartoon and GR20 highlighted with thicker coil, with marine for NTF2L and brown for GR20. The residues of NTF2L are shown as sticks, with yellow for acidic and magenta for arginine, and the arginine residues of GR20 are shown as lines. (B-C) BLI binding assay of full-length G3BP1 and GR20 carrying the single mutations of (B) R32K with K_D = 110 ± 1 nM and (C) R17K with K_D = 134 ± 1 nM in the NTF2L domain.

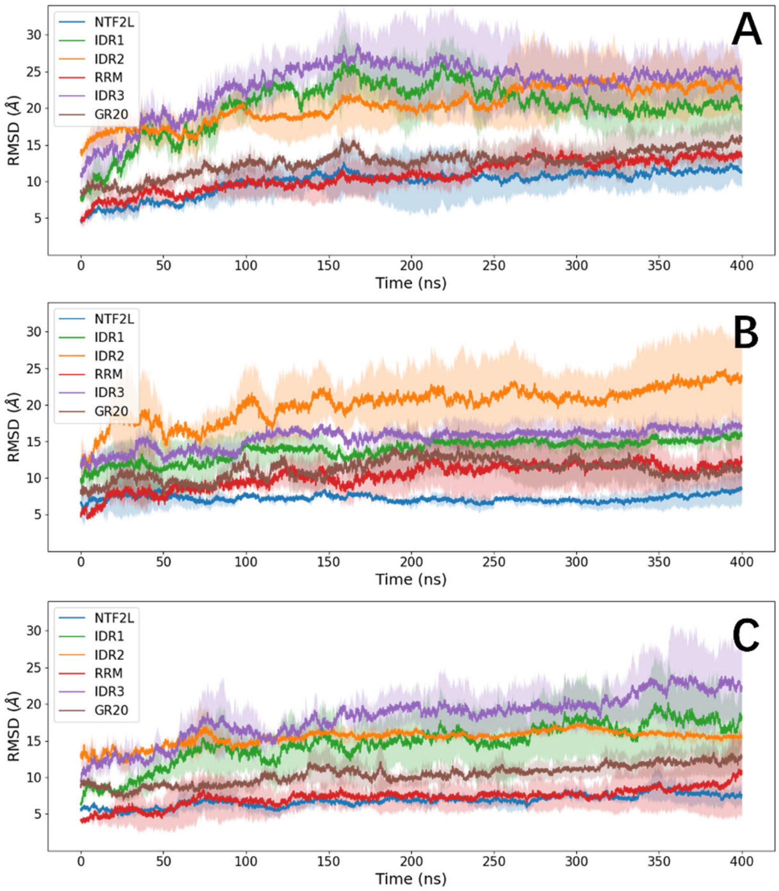

Figure S3. Heavy-atom root-mean-square deviation (RMSD) relative to the initial AFEX model over MD simulation time. The trajectories were sampled at pressure of 1 bar and temperature of (A) 500 K, (B) 420 K, and (C) 350 K. Transparent shading represents the range of values from three independent simulation replicates.

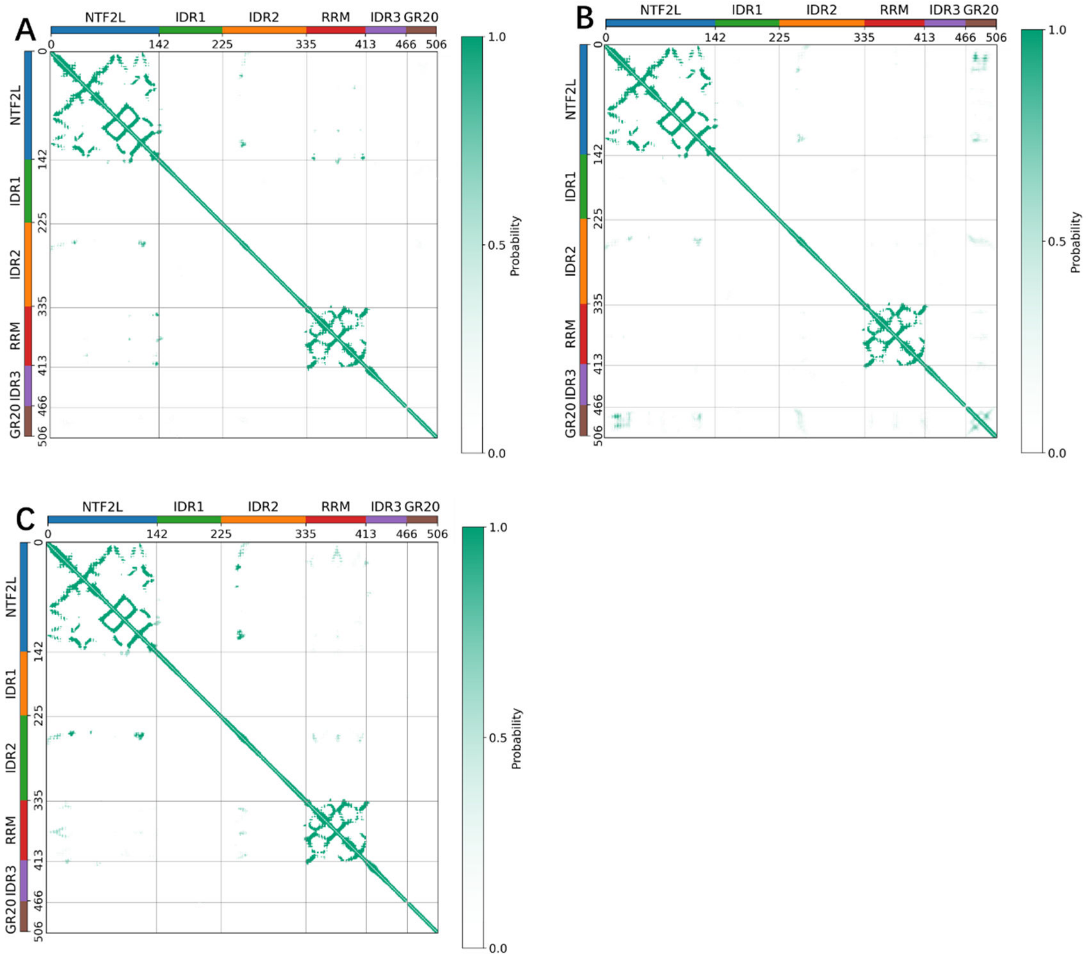

Figure S4. Residue contact map represented by pairwise Cα distance of AF models. (A-C) G3BP1-GR20 complexes generated using AlphaFold v2.2 (A), v2.3 (B), and v3 (C). The probability is counted from all relaxed output structures.

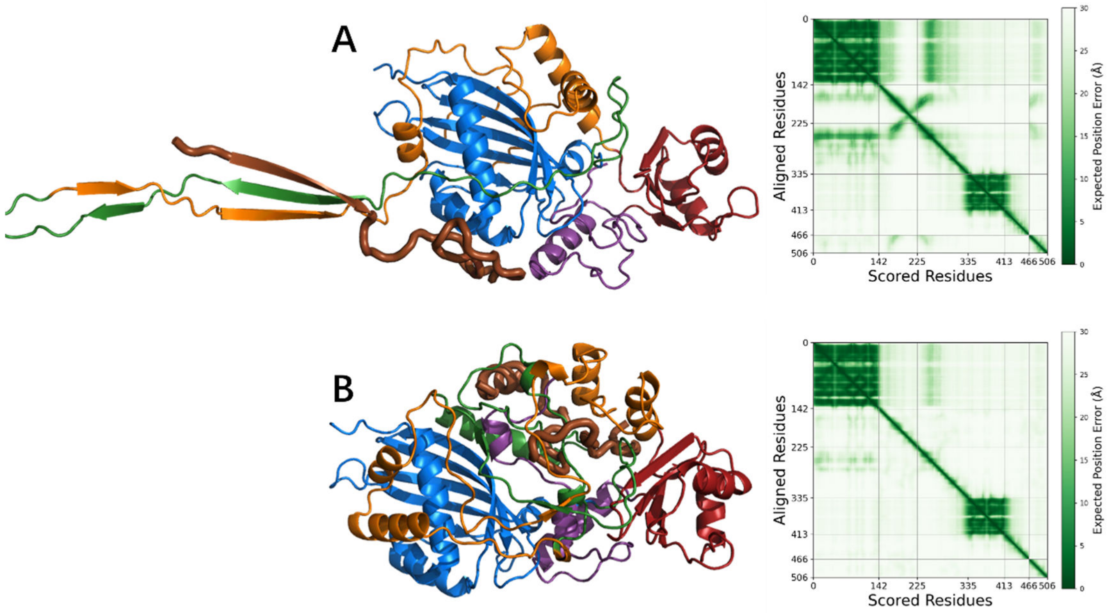

Figure S5. G3BP1-GR20 models predicted using Boltz-2. Predicted structures and corresponding PAE plots for the first output model generated with (A) Boltz-2 v.1.4.9 and (B) Boltz-2 v.2.2.0. Structural domains are colored as follows: NTF2L (marine), IDR1 (green), IDR2 (orange), RRM (crimson), IDR3 (violet), and GR20 (brown). GR20 is additionally highlighted with a thicker cartoon representation.
